# Modelling lipid rafts formation through chemo-mechanical interplay triggered by receptor–ligand binding

**DOI:** 10.1007/s10237-023-01787-2

**Published:** 2023-12-07

**Authors:** Chiara Bernard, Angelo Rosario Carotenuto, Nicola Maria Pugno, Massimiliano Fraldi, Luca Deseri

**Affiliations:** 1 Department of Civil, Environmental and Mechanical Engineering, University of Trento, Trento, Italy; 2 Department of Structures for Engineering and Architecture, University of Naples “Federico II”, Naples, Italy; 3 Laboratory of Integrated Mechanics and Imaging for Testing and Simulation (LIMITS), University of Naples “Federico II”, Naples, Italy; 4 Laboratory for Bioinspired, Bionic, Nano, Meta Materials and Mechanics, University of Trento, Trento, Italy; 5 School of Engineering and Materials Science, Queen Mary University of London, London, UK; 6 Department of Mechanical Engineering and Material Sciences, MEMS-SSoE, University of Pittsburgh, Pittsburgh, USA; 7 Department of Civil and Environmental Engineering, Carnegie Mellon University, Pittsburgh, USA; 8 Department of Mechanical Engineering, Carnegie Mellon University, Pittsburgh, USA; 9 Département de Physique, LPENS, École Normale Supérieure-PSL, Paris, France

**Keywords:** Ligand–receptor binding, Lipid rafts, GPCRs, Mechanobiology, Cahn–Hilliard, Cell membrane

## Abstract

Cell membranes, mediator of many biological mechanisms from adhesion and metabolism up to mutation and infection, are highly dynamic and heterogeneous environments exhibiting a strong coupling between biochemical events and structural re-organisation. This involves conformational changes induced, at lower scales, by lipid order transitions and by the micro-mechanical interplay of lipids with transmembrane proteins and molecular diffusion. Particular attention is focused on lipid rafts, ordered lipid microdomains rich of signalling proteins, that co-localise to enhance substance trafficking and activate different intracellular biochemical pathways. In this framework, the theoretical modelling of the dynamic clustering of lipid rafts implies a full multiphysics coupling between the kinetics of phase changes and the mechanical work performed by transmembrane proteins on lipids, involving the bilayer elasticity. This mechanism produces complex interspecific dynamics in which membrane stresses and chemical potentials do compete by determining different morphological arrangements, alteration in diffusive walkways and coalescence phenomena, with a consequent influence on both signalling potential and intracellular processes. Therefore, after identifying the leading chemo-mechanical interactions, the present work investigates from a modelling perspective the spatio-temporal evolution of raft domains to theoretically explain co-localisation and synergy between proteins’ activation and raft formation, by coupling diffusive and mechanical phenomena to observe different morphological patterns and clustering of ordered lipids. This could help to gain new insights into the remodelling of cell membranes and could potentially suggest mechanically based strategies to control their selectivity, by orienting intracellular functions and mechanotransduction.

## Introduction

The study of the lipid membranes plays a pivotal role in the understanding of many biological mechanisms occurring in cell physiology (Simons and Ikonen 1997; Simons and Toomre 2000; Simons and Ehehalt 2002). It is well known that the lipid bilayer is a selective-permeable membrane involving a large number of molecular species regulating the transport of nutrients and ions inside and outside the cell (Nagle and Tristram-Nagle 2000; Bloom et al. 1991). As well known, cell membranes are composed of amphiphilic phospholipids with a polar head group and a hydrophobic tails (Alberts et al. 2007). The re-configuration of the latter ones, together with the corresponding change in stereometric packing of the lipids, is responsible for the bilayer phase transition from a liquid-disordered phase to a liquid-ordered one also called the *raft phase* (Brown and London 1998). Lipid tails organisation thus also implies different membrane composition and thickness. In fact, in the raft phase functional microdomains, composed by densely packed sphingolipids and cholesterol, form compact isles named *lipid rafts*, in which different types of transmembrane protein complexes are assembled and segregated (Semrau and Schmidt 2009; Toulmay and Prinz 2013). The importance of unveiling the mechanobiology behind lipid rafts formation can support the understanding of a vast variety of membrane-mediated processes through which cells communicate with the extracellular environment. Actually, since the lipid raft hypothesis has been demonstrated thanks to the advances in imaging techniques and probing microscopy (Simons and Ikonen 1997; Baumgart et al. 2003; Leslie 2011; Gaus et al. 2003), their role in cell physiology has been deeply investigated. In the recent years, increasing evidences have shown indeed that lipid rafts serve as highly organised communication hubs for cell membranes by favouring the assembly of the most of proteins involved in cell signalling and trafficking (Helms and Zurzolo 2004; Barnett-Norris et al. 2005; Koyama-Honda et al. 2020; Ouweneel et al. 2020). This establishes a phenomenon of co-localisation, by means of which the most of transmembrane proteins are mainly clustered in correspondence of lipid rafts, including signalling receptors—the G-protein coupled receptors (GPCRs) and the receptor tyrosine kinase (RTK)—as well as resident proteins like glycosylphosphatidylinositol (GPI) anchored proteins and caveolin, but also cytoskeletal and adhesion proteins such as integrins and cadherins that participate to cell structural re-arrangement (Phillips et al. 2009; Simons and Sampaio 2011; Lorent et al. 2017). It is widely recognised that this co-localisation and coalescence of nano-domains into mesoscopic ones, due to the interplay of receptor–ligand binding, plays a key role in promoting cell-cell adhesion (Li et al. 2021, 2022). In particular, the way in which receptor–ligand binding affects the distribution of lipid rafts and the heterogeneity of the cell membranes still remains an open issue. To this end, biological studies demonstrate the combined influence of receptor motility and the distribution of ligand molecules, as well as their degree of interaction with lipid components (Li et al. 2022). Furthermore, the recent results report how the thermodynamic coupling of activated protein to ordered lipid phases is fundamental for the functional organisation of lipid membranes (Wang et al. 2023). Lipid phase separation is indeed enhanced and stabilised by local protein condensation and resident macro-molecules such as cholesterol. In turn, co-localisation on rafts promotes signal transmission by favouring the biochemical interaction among the nearby proteins that regulate the flux of substances. Given their prominent position in triggering cell functions, lipid rafts thus play a primary role also in cell immune response and in several diseases (Wang et al. 2023; Varshney et al. 2016; Shao et al. 2015; Hicks et al. 2012; Sorci-Thomas and Thomas 2016). Among these, the role of lipid rafts as signalling platform for bio-markers is involved in cancer development and progression (Beloribi-Djefaflia et al. 2016; Mollinedo and Gajate 2020; Murai 2012; Zhang et al. 2022). Evidences show that many receptors from the GPCRs’ family appear over-expressed in malignant cells and affect phenotypic differentiation and cancer degeneration of different types of cells (Staubach and Hanisch 2011). Also, lipid rafts are indirectly involved in many cell internalisation mechanisms, since they host macromolecular architectures responsible for endocytosis and exocytosis. This implies that they serve as an entry-port for many species of viruses including Poliovirus and Coronaviruses, by representing the sight of first contact between cell and virus (Upla et al. 2009; Kulkarni et al. 2022; Lu et al. 2008; Luo et al. 2008; Chazal and Gerlier 2003; Ripa et al. 2021; Sorice et al. 2021).

**Fig. 1:**
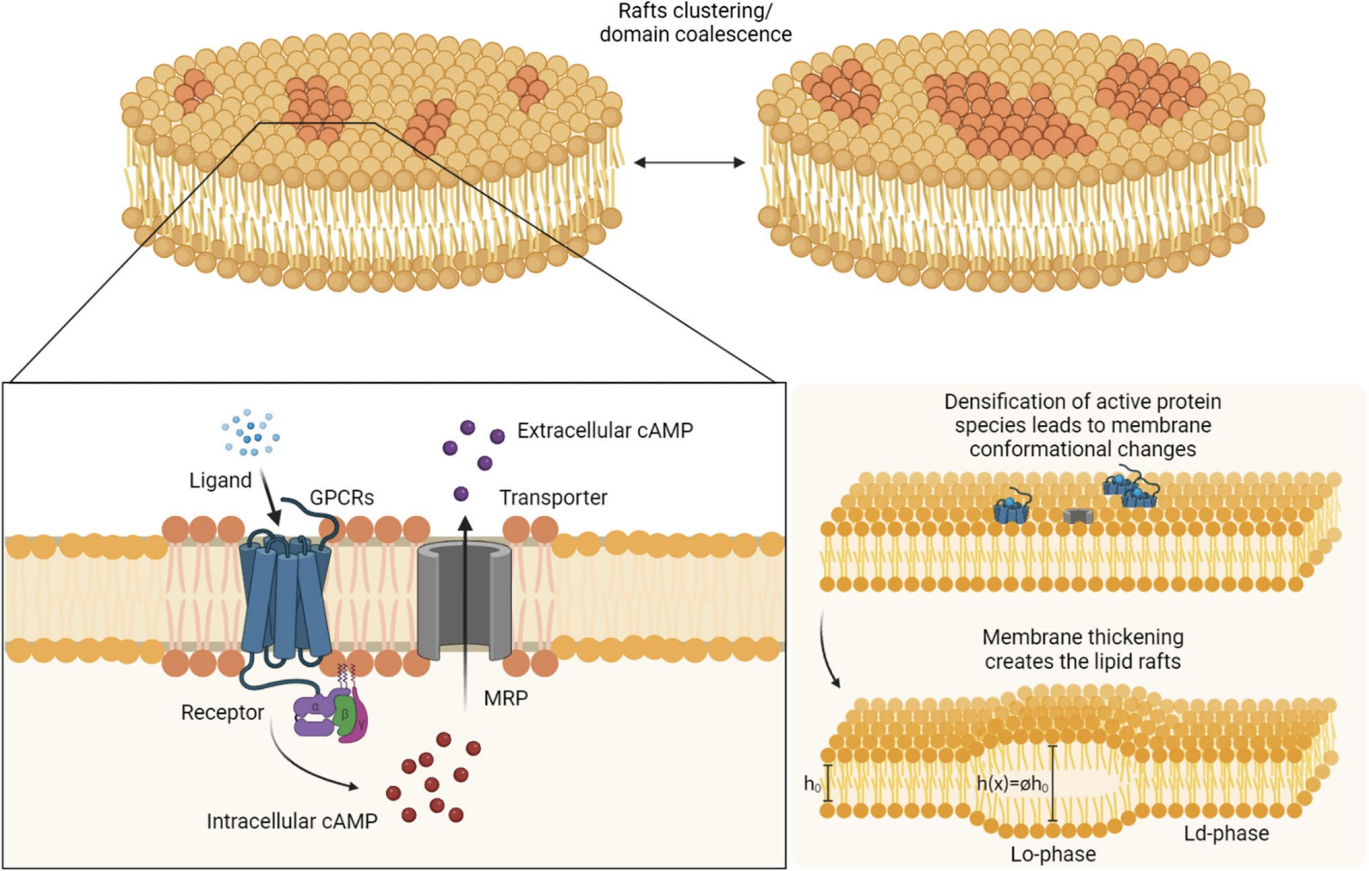
Schematic representation of transmembrane proteins activation and the consequent membrane remodelling. The process of densification of proteins species give rise to the nucleation and coalescence of such active domains. Also, this drives the system towards conformational changes and thickness variation

The vast involvement of lipid rafts in ruling several cellular processes in homeostatic and abnormal conditions requires the necessity of a broader comprehension of the mechanisms underlying the formation and clustering of raft domains in co-evolution with the dynamics of active transmembrane proteins. In particular, one of the most abundant signalling protein species in human cells is the afore-mentioned GPCRs family (Harmar 2001). As it turns out, GPCRs activation is due to ligand binding and it does involve rigid body transformations of their morphology by so directly interacting with the membrane in situ (Rosenbaum et al. 2009; Li et al. 2023). It is indeed confirmed that GPCRs exhibit remarkable conformational rearrangements passing from inactive to active states (Gurevich and Gurevich 2017), thus influencing structural changes in the surrounding lipids (Samama et al. 1993; Manglik et al. 2015). Above all the receptors included in the GPCRs family, the $\beta _2-$adrenergic receptors ($\beta _2$ARs) are the most studied ones given their leading role as target receptors for drug design (Daly and McGrath 2011). In fact, $\beta _2$AR binds to the hormone epinephrine that plays an important function in most of the human organs (Manna et al. 2016), literature evidences showing that this ligand binding activates $\beta _2$ARs which result then to localise on lipid rafts (Chini and Parenti 2004; Van Anthony et al. 2016; Ostrom and Insel 2004; Wright et al. 2015). Here, they respond to a vast class of environmental stimuli that are translated into a cascade of biochemical reactions through secondary messengers. For instance, this is the case of cyclic adenosine monophosphate (cAMP), whose production in the intracellular environment is triggered by such receptors activation. This is fundamental to mediate intracellular physiological functions, including proliferation, differentiation and gene expression (Calebiro et al. 2009; Beavo and Brunton 2002). However, in order to avoid the over-expression of G-receptors both temporally and spatially and in turn restore cellular homeostasis, desensitisation of GPCRs is needed through exposure to an antagonist. This action is carried out by multidrug-associated resistance proteins (MRPs), which exerts a proper down-regulating feedback for inhibiting receptor responsiveness and promotes the efflux of cAMP to the extracellular space by preventing intracellular excessive accumulation (Reid et al. 2003; Chen et al. 2001; Wielinga et al. 2003; Biondi et al. 2006; Lunghi et al. 2007; Biondi et al. 2010) (see Fig. 1). Therefore, the cell membrane presents intra and extracellular trafficking of various species, which induces morphological transitions of the transmembrane proteins regulating the cell response to external and endogenous stimuli (Cevc and Richardsen 1999), with a consequent effect on surrounding lipids in terms of conformational adaptation. Such strong interaction of the macro-molecules re-configuration and mobility with lipid ordering inevitably calls into play a connection between biochemical and physical agents (Jacobson et al. 2007). Actually, the established knowledge in biology according to which ligand–receptor complexes form only by providing conformational changes of the active receptors has been recently confirmed (Frei et al. 2020).

For this reason, several approaches have explored the correlation between lipid phase changes and the mechanical properties of biomembranes. Recently, in the work by (Carotenuto et al. 2020), the governing principles behind the synergy between protein activation and membrane energetics have been analysed by means of mechanical models enriched with multicomponent diffusive–reactive systems. In spite of the high heterogeneity of proteins in raft micro-environment, some leading spatio-temporal phenomena and interspecific cooperative/agonist mechanisms among them have been traced back by following GPCRs and by accounting for their chemical feedback terms of interspecific nature (Fraldi and Carotenuto 2018; Carotenuto et al. 2018) as well as the cross-talk between membrane elasticity and species momentum. This has been done through the introduction of suitable coupling terms that translate, at the membrane scale, the micro-mechanical interaction between protein dynamics and membrane adaptation. The deformation of the lipid bilayer is then the result of interspecific chemical reactions coupled with structural changes that depend on the membrane properties. Also, a fully coupled thermodynamic framework in which tracking both the chemical agonists across the lipid bilayer and the membrane material response can be simultaneously considered based on such paper (Carotenuto et al. 2020). There, the above-discussed communication mechanisms between GPCRs and MRPs have been introduced in terms of equivalent densities of active species involved in ligand binding of ubiquitous transmembrane proteins at the membrane scale. In particular, the explicit interplay among the lipid bilayer elasticity and density changes of the active species, generating a kinematically independent remodelling, has been explored. The kinetics of the species involved has been predicted through a reaction-diffusion system of equations in which interacting interspecific terms turned out to get coupled with the thickness stretching of the membrane. The outcomes of this approach aim at providing evidence that active receptors prefer to cluster on lipid rafts also enhancing their formation. Such a study, among other results, does allow for the first (mechanically based) explanation and prediction of why the higher-density regions of active receptors are lipid rafts. Nevertheless, some phenomena such as nucleation and coalescence of these rafts were not included in the model developed in Carotenuto et al. (2020). Although rafts are primarily geometrical phases that relate to thickness changes, the fact that higher densities of active receptors are found on lipid rafts suggests an alternative way to keep track of them. Therefore, the main focus of the presented model is to enrich, in a two-dimensional framework, the aforementioned study through a Cahn–Hilliard-like diffusion (Cahn and Hilliard 1958; Gurtin 1996) for the active species involved in the proposed mechanobiology. In fact, a Cahn–Hilliard approach is commonly utilised for describing and modelling the evolution of phase separations in a vast class of problems (Snyder et al. 1983; Zhou and Wang 2007; Agosti et al. 2017; Khodadadian et al. 2019; Miehe et al. 2014) and it results particularly suitable for tracing back the variation of lipid membrane composition. This is done in spite of the fact that phase changes are purely geometrical in this case. Furthermore, balance of forces regulates such situations by resorting to uncoupled (Zhiliakov et al. 2021; Witkowski et al. 2012) and extended diffusive models (Garcke et al. 2016). For instance, in the latter work, the dynamics of the order parameters mapping the lipid domain to follow the dynamics of the biological membrane have been analysed in correlation with the influence of cholesterol bulk diffusion. In this way, it has been shown the emergence of raft-like structures in the non-equilibrium case, as opposed to the sole macro-domains survival, for the long-time evolution, in the equilibrium case. However, some leading effects provided by the interaction with the elasticity of membrane influencing species motility and activation as well as the pivotal role played by competitive protein species in driving raft formation and desensitisation-induced annihilation at long time, have been still neglected. Solid literature works have been dedicated to the general characterisation of Cahn–Hilliard dynamics in fluid-like environments by means of both analytical methods and computational frameworks based on phase-field approaches (Lowengrub and Truskinovsky 1998; Gomez and Zee 2017), although the order parameter classically involved in Cahn–Hilliard-type problems has been rarely connected to the underlying (bio)-physics. Actually, there is still no approach that examines the influence of membrane areal changes (or, alternatively, of their thickness variations) on the growth and coalescence of active protein domains yet.

Therefore, based on the newly obtained formulation (Carotenuto et al. 2020) and on the well-established ground of Cahn–Hilliard models, the focus of the present paper is to explore the inherent synergistic coupling of transmembrane species’ coalescence dynamics. This is enriched by reactive interspecific feedback and the space-time evolution of lipid domains and their interaction mediated by the membrane mechanical response within a nonlinearly elastic environment. Indeed, it is shown that the effective cross-talk between mechanical and protein fields turns out to be a not negligible environmental factor in orienting protein densification and spatio-temporal arrangement. Furthermore, this has a direct impact on the dynamic remodelling of membrane properties in terms of inhomogeneous dilation of membrane thickness and heterogenisation of the associated mechanical response. In order to highlight these particular aspects and put focus on the driving role of nucleation and coalescence, numerical simulations in finite element analyses (FEA) have been carried out by using a two-dimensional continuum model and by considering a neo-Hookean membrane in plane stress conditions (as also adopted in some works relating to the investigation of biomembranes properties (Bavi et al. 2014; Mahata et al. 2022; Carotenuto et al. 2021)), thereby neglecting the self-capability of lipid bilayers to re-organise themselves in ordered and disordered patches even in absence of proteins (Deseri and Zurlo 2013; Deseri et al. 2008). This, at least to some extent, could potentially affect the corresponding bilayer thickening and further enrich the spatio-temporal multiphysics competition.

## Coupled modelling of lipid bilayer phase transition

In this section, we summarise the mechanics of the cell membrane by describing the way in which the mechanical behaviour of the lipid bilayer is coupled to Cahn–Hilliard-like interspecific equations that govern the spatio-temporal evolution of lipid phase. Indeed, under physiological conditions, lipids typically undergo an order–disorder phase transition, manifesting as a thickness change that characterises the raft phase. In the latter, lipids get straightened and, hence, ordered while in the surrounding thinner zones the constituents are more disordered. Models analysing the mechanical behaviour of lipid membranes undergoing phase transitions are typically based on nonlinear hyperelastic laws owing to predict their deformations. From a mechanobiological perspective, it has been demonstrated how phenomena underlying lipid phase transition and membrane remodelling, within a heterogeneous context, can be traced back by accounting for fully coupled relationships among macroscopic deformation, conformational changes of the transmembrane receptors populating such systems, and biochemical events triggered by external ligands chemically affine to the transmembrane proteins just mentioned (Carotenuto et al. 2021, 2023). The latter paper shows how to account for the known fact that such ligands activate their affine receptors by causing appropriate changes in their configuration while confined by the surrounding lipids. Thus, unlike available pure mechanical descriptions of the lipid bilayers (see e.g. Deseri and Zurlo (2013); Maleki et al. (2013)) or purely diffusive approaches neglecting the influence of micro-mechanical environmental stimuli, a multiphysics approach is proposed by coupling the kinematics and the kinetics of phase changes and also the conformational changes of the active receptors.

Following the previous work on lipid bilayers in Carotenuto et al. (2020), we analyse two-dimensional systems that, while experiencing phase separation by positioning clusters of ordered lipids on thicker islands, can explicitly manifest raft coarsening and nucleation. The phenomenon is still governed by a coupling of the remodelling and the energetics of the membrane given by the active proteins involved in the process, exactly as obtained in Carotenuto et al. (2020). Nonetheless, some simplifications on the elasticity of the lipid membrane will be performed in favour of the introduction of kinetics accounting for nucleation and merging of rafts. Therefore, here we utilise the whole novel mechanobiology strategy derived in Carotenuto et al. (2020) in spite of the fact that the nonconvexity of the membrane energy (in the local areal change measure) is neglected, thereby not considering the spontaneous trend of lipids to re-organise themselves in coexisting phases even in the case of artificially made bilayers. That simplification is in favour of focusing on the effects produced by the receptors and transporters on raft formation and coalescence, thereby including a Cahn–Hilliard-type dynamics on the species evolution. That is for keeping track of the nucleation and evolution of lipid islands, following the corroborated idea that active receptors prefer to cluster on lipid rafts (see e.g. Carotenuto et al. (2020) and references cited therein).

### Kinematics of the membrane

From the mechanobiological standpoint, the cell membrane deforms by increasing the density of the lipids which align themselves with the surface normal. For modelling such behaviour, the lipid membrane is assumed to be a quasi-incompressible hyperelastic surface for which the areal stretch and thickness locally vary according to the corresponding changes of the lipid order. In spite of those geometrical features, entailing thinning and thickening of the bilayer, many authors primarily looked at ways for penalising curvature changes only. In this respect, the classical Canham–Helfrich theory has been the most adopted one to represent lipid bilayer membranes (Canham 1970; Helfrich 1973). Indeed, the *Helfrich free energy*, a generalisation of the Kirchhoff-Love strain energy for thin shells undergoing large curvature changes, has been accepted in biophysics as the keystone free energy density for the study of elastic surfaces in the case of pure bending (Safran 2018; Deserno and Bickel 2003; Rangamani et al. 2021). However, through this approach, there is no information about the changes in thickness across the membrane. Hence the necessity of a different surface energy density that can keep track of thickness variations becomes manifest (Zurlo 2006; Deseri et al. 2008; Trejo and Ben Amar 2011). In this regard, in Deseri et al. (2008) a dimensionally reduced energetics is presented. It correlates lipid phase transition, curvature changes, membrane remodelling (directly connected to the macroscopic deformation of the membrane), and chemical composition. However, while the bending regime of the membrane is known to be highly involved in many cellular processes such as endocytosis (Gao et al. 2005), there is evidence that the phenomenon of lipid condensation is mainly driven by the sub-macroscopic dynamics of surface proteins and physically affected more by line tension rather than by macroscopic curvature effects (Witkowski et al. 2012). Therefore, within the framework of membrane elasticity, the main hypotheses made in Deseri and Zurlo (2013), Zurlo (2006), Deseri et al. (2008) are here introduced: the membrane is assumed to be flat and its kinematics is confined in the class of normal preserving deformations. In the geometrical setting, the natural configuration of the membrane $\mathcal {B}_0$ is divided in a two-dimensional system $\textbf{x}= x {} {\textbf {e}}_1+ y {} {\textbf {e}}_2$ and the thickness *z*, so that the material particles $\varvec{ x }\,\in \, \mathcal {B}_0$ are described as $\varvec{ x } = \textbf{x} + z\mathbf {e_3}$.

On these bases, the displacement field reads as follows:

1 $$\begin{aligned} {\textbf {u}}\left( x,y,z,t\right) =\left[ u_1\left( x,y,t\right) ,u_2\left( x,y,t\right) ,\left( \phi \left( x,y,t\right) -1\right) z\right] . \end{aligned}$$

Therefore, the deformation gradient of the elastic membrane reads:

2 $$\begin{aligned} \textbf{F} = \textbf{I} + \nabla \textbf{u} = \begin{bmatrix} 1+u_{1,x} &{} u_{1,y} &{} 0 \\ u_{2,x} &{} 1+u_{2,y} &{} 0 \\ z \phi _{,x} &{} z \phi _{,y} &{} \phi \end{bmatrix} \end{aligned}$$

with the function $\phi \left( x,y,t\right)$ representing the thickness stretch in the direction $\textbf{e}_3$. By restricting the problem to the mid-plane of the membrane (see e.g. Deseri and Zurlo (2013); Zurlo (2006); Deseri et al. (2008)) and by accounting for a volumetric incompressibility constraint, a condition on the determinant of $\textbf{F}$ at $z=0$ is defined:

3 $$\begin{aligned} J = J_0 \phi = 1, \end{aligned}$$

namely $\phi=\frac{1}{J_0}$, where $J_0$ denotes the areal stretch of planes perpendicular to $\textbf{e}_3$, i.e. $J_0=\det \,\textbf{F}_0$ where $\textbf{F}_0=\textbf{F}(x,y,0,t)-\phi (\textbf{e}_3\otimes \textbf{e}_3)$ (in the sequel $\textbf{F}(x,y,0,t)$ will be indicated with $\textbf{F}$ and the dependence on *z* will be suppressed). In the light of these considerations, we will assume in what follows an energy density that depends on the considered stretch components –i.e. of $\textbf{F}_0$ and $\phi$– as well as on the density of the protein species $n_i$ in order to set up the equations governing the coupled dynamics of the problem.

### Constitutive assumptions for the lipid bilayer

The energetics of the system at hand is assumed to be governed by the Helmholtz free energy density $\mathcal {W}\left( \textbf{F},n_i, \nabla n_i, \phi \right)$, in which the term $\nabla n_i$ is introduced to account for potential boundary layers where the densities of the active species may gradually vary. This actually happens in coalescence phenomena for such species. This is an energy per unit area in the active configuration, i.e. where lipids re-organise themselves due to the activation of GPCRs influencing membrane thickness. In particular, an additive decomposition is assumed for $\mathcal {W}$ to represent both the contributions given by the potential associated with the hyperelastic energy of the membrane, namely $\mathcal {W}_{\text {hyp}}$ , and $\mathcal {W}_{n_i}$ related to the transmembrane proteins:

4 $$\begin{aligned} \mathcal {W}=\mathcal {W}_{\text {hyp}}\left( \textbf{F}\right) + \mathcal {W}_{n_i}\left( n_i, \nabla n_i, \phi \right) . \end{aligned}$$

**Fig. 2:**
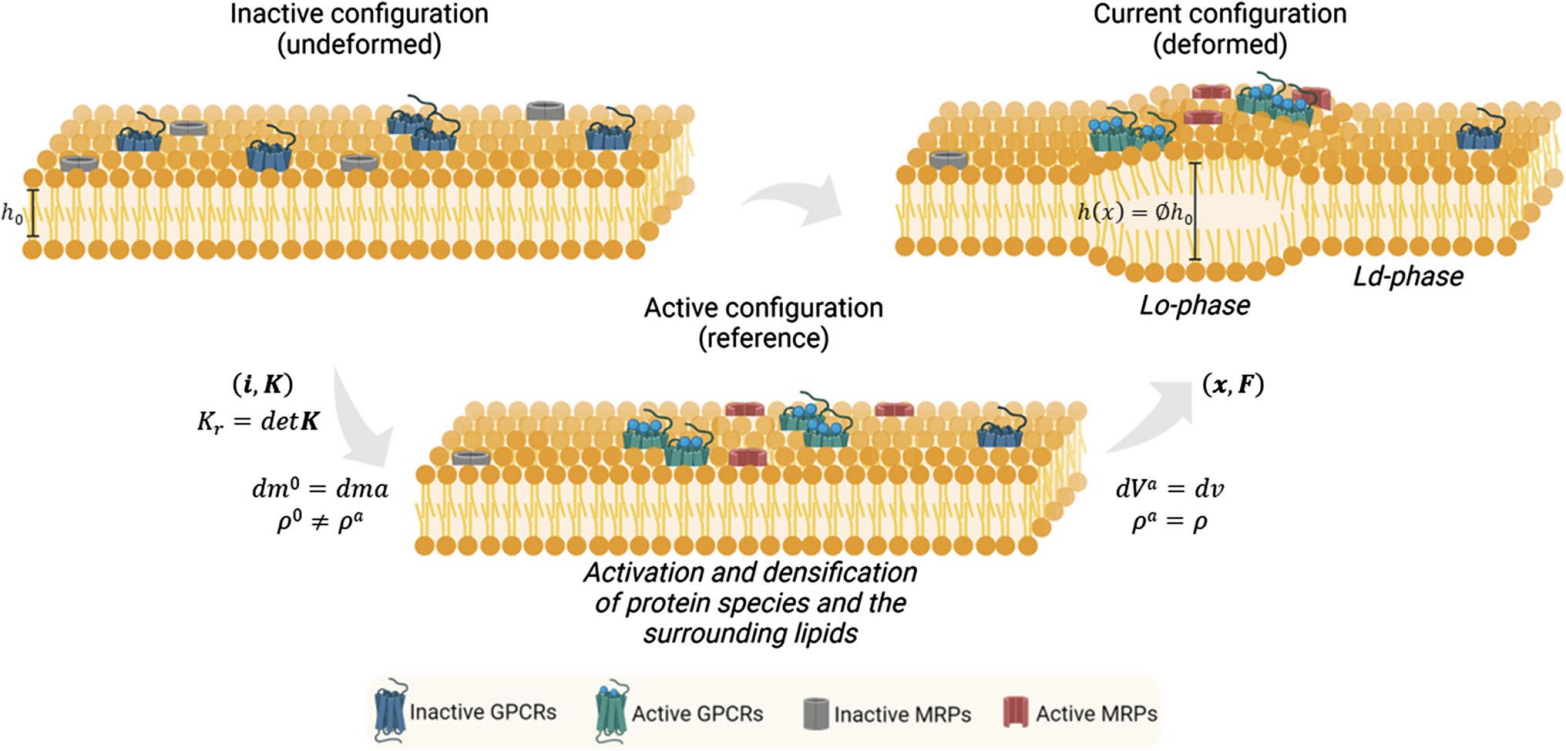
Protein activation and membrane remodelling. Transmembrane protein activation and densification leads the system towards an active configuration where lipid re-organise their tails which, in turn, create thicker zones thus deforming the membrane’s structure. Hence, active species conformational changes induce the remodelling of the lipid membrane where rafts are formed. This process is modelled through the theory of *Structured Deformations* (Deseri and Owen 2003, 2010, 2019, 2015; Palumbo et al. 2018), a multiscale geometric framework that allows for distinguishing between the active reference configuration and the current deformed configuration. The former is characterised by the term $K_r$ standing for the change in volume induced by disarrangements that are here caused by the sub-macroscopic remodelling. Then, the pair $\left( {\textbf {x}},{\textbf {F}}\right)$ represents the classical deformation occurring from the intermediate global configuration to the current one

Unlike classical chemical free energies, here the contribution $\mathcal {W}_{n_i}$ is explicitly dependent on the stretch parameter $\phi$. This allows for considering the direct influence that species evolution has on membrane deformation and, vice versa, on how membrane thickening can kindle additional chemo-mechanical stimuli for species aggregation and steer the co-localisation. This comes from micro-mechanical considerations characterising the work exerted by the lateral pressure arising in the lipid bilayers to mediate the conformational changes of the active species (Carotenuto et al. 2020). In deriving suitable constitutive assumptions for the model at hand, a configurational term arises due to sub-macroscopic activation of protein loci, which induce microstructural changes related to the local density variation before experiencing macroscopic deformation. In particular, by referring to the scheme in Fig. 2, based on the theory of Structured Deformations (see e.g. Del Piero and Owen (1993); Deseri and Owen (2003, 2010, 2019, 2015); Palumbo et al. (2018), and references cited therein), it is considered that the reference membrane in the (inactive) virgin configuration $\mathcal {B}_0$ (characterised by a species density $n_i^0$) is, first, mapped onto a macroscopically identical intermediate (reference) configuration called $\mathcal {B}_a$. There, at each point, some of the inactive proteins are activated by forming ligand binding complexes by kindling subsequent membrane adaptation towards the actual (deformed) configuration $\mathcal {B}$, in which the emergence of lipid rafts occurs as a deformative effect. More specifically, during the activation of proteins, sub-macroscopic re-arrangement of such species and surrounding lipids are witnessed by a configurational Jacobian $K_r$ expressing the local densification of the species. An equation for such a field can be derived by considering that the mass of the system is not varying so that the condition ${\text {d}}m^0={\text {d}}m^a$ gives (see Carotenuto et al. (2020) for details):

5 $$\begin{aligned} K_r=\frac{{\text {d}}V^a}{{\text {d}}V^0}=\frac{\rho ^0}{\rho ^a}= \frac{1+k_u \left( 1+ \Sigma _i n_i^0 \Delta _A\right) }{1+k_u \left( 1+ \Sigma _i n_i \Delta _A\right) }. \end{aligned}$$

Here, $k_u$ denotes the total areal fraction of the protein in their inactive state, and $\Delta _A$ represents an estimate of the relative change in area of the proteins on passing from the inactive to the active configuration. It is worth noting that the remodelling term $K_r$ is related to the conformational changes of the receptors during ligand binding across the membrane occurring at the sub-macroscopic scale. Since the membrane experiences multiple configurations through a first activation path and a subsequent classical (incompressible) deformation, the three-dimensional free energy density $w^*$ in the actual (deformed) state can be expressed as corresponding energy density contributions both in the natural (active) configuration as well as in the virgin state, say $\mathcal {W}_a^*$ and $\mathcal {W}^*$, respectively. These ones will be associated with each other through the following relations:

6 $$\begin{aligned} \int _V\, w^*{\text {d}}v = \int _{V^a}\, \mathcal {W}_a^*{\text {d}}V^a =\int _{V^0}\,K_r\, \mathcal {W}^*{\text {d}}V^0. \end{aligned}$$

Moreover, because of the thinness of the cell membrane relative to its in-plane sizes, a dimension reduction procedure returns the above-mentioned energy density $\mathcal {W}$ per unit area in the virgin configuration:

7 $$\begin{aligned} \mathcal {W}=\int _{-h_0/2}^{h_0/2}\, \mathcal {W}^*{\text {d}}z=h_0\,\mathcal {W}^*. \end{aligned}$$

Hence, in order to establish a thermodynamically consistent framework upon which to evaluate the chemo-mechanical coupling between transmembrane protein dynamics and membrane elasticity, the energy-entropy imbalance is written with respect to the inactive (virgin) configuration to obtain the constitutive set of the problem. This provides the mechanical power produced by the nominal stress tensor $\textbf{P}^*$ and the chemical contributions involving species’ reference flux $\textbf{Q}_i$ and source terms $\Gamma _i$, which are driven by scalar chemical potentials $\mu _i^*$ as well as additional terms of entropic nature. The dissipation inequality thus reads as follows:

8 $$\begin{aligned}{} & {} \int _{V^0} \textbf{P}^*:\dot{\textbf{F}}\,{\text {d}}V^0 - \int _{S^0} \mu _i^*\,\textbf{Q}_i\cdot \textbf{N}\,{\text {d}}S^0 + \int _{V^0}\mu _i^*\,\Gamma _i \,{\text {d}}V^0 \nonumber \\{} & {} \quad + \int _{V^0}\,\mathcal {W}^*\,\dot{K}_r\,{\text {d}}V^0 \ge \, \frac{{\text {d}}}{{\text {d}}t}\int _{V_0} K_r\,\mathcal {W}^*\,{\text {d}}V^0 \nonumber \\{} & {} \quad + \int _{V_0} \textbf{Q}_i\cdot {\varvec{{\Lambda }}}_i\cdot \textbf{Q}_i\,{\text {d}}V^0, \end{aligned}$$

where the subscript *i* denotes summation over the species set and $V^0=h_0\,\Omega$ is the undeformed volume. With regard to the entropic terms, the third term on the left-hand side of the equation represents the power contribution per unit mass production due to the remodelling rate (see e.g. Lubarda and Hoger (2002)), while the last term on the right-hand side of (8) models the dissipation due to species transport mediated by the friction coefficient matrices ${\varvec{{\Lambda }}}_i$. On account of equation (4), after few steps, inequality (8) gives:

9 $$\begin{aligned}{} & {} \int _{V^0} \left[ \textbf{P}^*:\dot{\textbf{F}} -\textbf{Q}_i\cdot \nabla \,\mu _i^* -\textbf{Q}_i\cdot {\varvec{{\Lambda }}}_i\cdot \textbf{Q}_i \right. \nonumber \\{} & {} \quad \left. + \mu _i^*\left( -\nabla \cdot \textbf{Q}_i +\Gamma _i \right) \right] \,{\text {d}}V^0 \, \nonumber \\{} & {} \quad \ge \,\int _{V^0}\,K_r\left[ \frac{\partial \mathcal {W}^*}{\partial \textbf{F}}:\dot{\textbf{F}} + \left( \frac{\partial \mathcal {W}^*}{\partial \,n_i}-\nabla \cdot \frac{\partial \mathcal {W}^*}{\partial \,\nabla n_i}\right) \dot{n}_i\right] \,{\text {d}}V^0 \nonumber \\{} & {} \quad + \left[ K_r\,\hat{\textbf{N}}\cdot \frac{\partial \mathcal {W}^*}{\partial \,\nabla n_i}\dot{n}_i\right] _{\partial \,V^0}, \end{aligned}$$

The last term gives information about one of the boundary conditions, by requiring:

10 $$\begin{aligned} \left. \hat{\textbf{N}}\cdot \frac{\partial \mathcal {W}^*}{\partial \,\nabla n_i}\right| _{\partial V^0}=0. \end{aligned}$$

Also, the last parenthesis of the left-hand side of relation (9) can be related to the material rate of the species according to the generic mass balance equations for the *i*th species, that is:

11 $$\begin{aligned} \dot{n}_i=-\nabla \cdot \textbf{Q}_i +\Gamma _i. \end{aligned}$$

By further localising (9) and by collecting the other terms, one obtains the following reduced Clausius-Duhem inequality:

12 $$\begin{aligned}{} & {} \left[ \textbf{P}^*-K_r\,\frac{\partial \mathcal {W}^*}{\partial \textbf{F}}\right] :\dot{\textbf{F}} -\textbf{Q}_i\cdot \left[ \nabla \,\mu _i^* +{\varvec{{\Lambda }}}_i\cdot \textbf{Q}_i\right] \nonumber \\{} & {} \quad + \left[ \mu _i^*-K_r\,\left( \frac{\partial \mathcal {W}^*}{\partial \,n_i}-\nabla \cdot \frac{\partial \mathcal {W}^*}{\partial \,\nabla n_i}\right) \right] \dot{n}_i \ge 0. \end{aligned}$$

Upon applying the standard Coleman and Noll’s procedure, this allows for finding the following constitutive assumptions:

13 $$\begin{aligned}&\textbf{P}^*=K_r \, \frac{\partial \mathcal {W}^*}{\partial \textbf{F}} \quad \text {or} \quad \textbf{P}= K_r \, \frac{\partial \mathcal {W}}{\partial \textbf{F}}, \end{aligned}$$

14 $$\begin{aligned}\mu _i^*=&K_r \, \left( \frac{\partial \mathcal {W}^*}{\partial \,n_i}- \nabla \cdot \frac{\partial \mathcal {W}^*}{\partial \, \nabla n_i}\right) \quad \text {or} \nonumber \\\mu _i=&K_r \, \left( \frac{\partial \mathcal {W}}{\partial \,n_i}-\nabla \cdot \frac{\partial \mathcal {W}}{\partial \, \nabla n_i}\right) , \end{aligned}$$

15 $$\begin{aligned}&\textbf{Q}_i= -{\varvec{{\Lambda }}}_i^{-1}\cdot \nabla \, \mu _i^* \quad \text {or} \quad \textbf{Q}_i= -\textbf{L}_i\cdot \nabla \, \mu _i, \end{aligned}$$

where $\textbf{L}_i=h_0{\Lambda }_i^{-1}$ are the inverse of the friction matrices and thus represent the positive definite mobility coefficient of the species, which is assumed to have an isotropic structure.

#### Particularisation to the Cahn–Hilliard diffusion model and chemo-mechanical coupling

Many biological systems undergo phase separation processes leading to the formation of ordered microdomains with different sizes. These condensed zones are characterised by an increase in components concentration (Hyman et al. 2014). Typically, phase separation is mathematically described by the phase-field model through a diffusion equation for the species concentration (Chen 2002). In this way, it is possible to study the evolution of structures with complex morphologies, thus enabling a good comprehension of multicomponent systems. In particular, the Cahn–Hilliard is a phenomenological equation commonly used to describe two-phase biological systems (Cherfils et al. 2014). Among these, a particular attention is on cellular membrane that can be represented as a multicomponent bilayer mixture in which lipids and proteins coexist in two different phases (Heberle and Feigenson 2011). Indeed, lipid membranes experience phase transition between ordered and disorder phase and thus, lipid bilayer models should also include phase separation (Elson et al. 2010). Actually, an increase in the concentration of protein species has been observed on lipid rafts (Simons and Toomre 2000). The hypothesis behind raft formation is that the heterogeneity observed on lipid membranes can be addressed to the coexistence of phase-like domains (Hammond et al. 2005). The clustering of lipids leads to the initiation of most of cellular processes (Brown and London 1998; Edidin 2003; Chazal and Gerlier 2003), so it is necessary to introduce a phase separation diffusive model. On the other hand, protein microscopic rearrangements exert work on the surrounding membrane that, inevitably, calls into play the bilayer deformation and stress.

To model these phenomena, the potential associated with the transmembrane proteins provides a coupling between energy related to the chemical species plus a mechanical contribution linked to the protein activation-induced thickening of the membrane that is measured through the stretch $\phi$, i.e.:

16 $$\begin{aligned} \mathcal {W}_{n_i}\left( n_i, \nabla n_i, \phi \right) = \Psi _{n_i} - w_i\left( n_i-n_i^0\right) \left( \phi -1\right) , \end{aligned}$$

with $w_i$ representing a coupling parameter related to the strength of the mechanical work made by the *i*th protein species against the surrounding lipids. The term $\Psi _{n_i}$ is the canonical Ginzburg-Landau phase separation energy, typically used for representing the state of a bi-phase system close to its phase transition (Gurtin 1996). This potential depends on the phase $n_i$ and its gradient:

17 $$\begin{aligned} \Psi _{n_i} = \frac{1}{\epsilon }f(n_i)+\frac{\gamma }{2}\left| \nabla \left( n_i-n_i^0\right) \right| ^2, \end{aligned}$$

where $\epsilon$, $\gamma$ $>0$, while $\nabla \left( n_i-n_i^0\right)$ is so written to ensure condition (10) at the initial time. The function $f(n_i)$ is instead represented by a $double-well$ $potential$ usually taken according to the Flory-Huggins theory of mixtures (Berry et al. 2018). Here, the following suitable approximation of such term is considered:

18 $$\begin{aligned} f(n_i)=\frac{1}{4}n_i^2(1-n_i)^2, \end{aligned}$$

which displays a symmetric binary system with two local minima in 0 and 1, allowing for the coexistence of species. Gradients in chemical potentials guide the diffusive flux regulating differences in concentration of the protein fractions (Hyman et al. 2014). It is worth noticing that the standard double-well potential (18), with fixed minima associated to the active/inactive states, is here affected by the coupling term in (16) through the stretch $\phi$. In particular, this determines new non-symmetric minima positions by energetically favouring the active state in thickened membranes and, vice versa, the inactive one when the membrane contracts, so acting as a mechano-tactic signal establishing the influence of the surrounding mechanical environment and enhancing coalescence. Within this framework, the chemical potential associated to the Cahn–Hilliard diffusion dynamics can be written as:

19 $$\begin{aligned} \mu _i=& K_r \left[ -w_i \left( \phi -1\right) + \frac{1}{2 \epsilon }n_i\left( 1-n_i\right) \left( 1-2n_i\right) \right. \nonumber \\{} & {} \quad \left. - \nabla \cdot \gamma \nabla \left( n_i-n_i^0\right) \right] . \end{aligned}$$

As usual, $\nabla \mu _i$ represents the driving force generating species momentum in the mass balance of each protein population, mediated by a (scalar) diffusion mobility term $L_i$. Besides the classical Cahn–Hilliard-type chemical potential that kindle species coalescence, a cooperative mechanotaxis term is obtained through (19). This, according to Eq. (13), determines a flux $\propto w_i\nabla \phi$, which, *de facto*, represents the raft-induced attraction by which activating receptor and transporters tend to migrate towards the lipidic isle.

#### Interspecific equations for protein species

The generic mass balance Eq. 11) for each species is written in terms of the material time derivative, the material flux, and the evolutionary term of each protein fraction. In particular, the flux vector $\textbf{Q}_i=-L_i\,\nabla \mu _i$ can be calculated according to the chemical potential derived in Eq. (19). Differently from classical Cahn–Hilliard dynamics, the mass balances also provide nonzero interspecific rates $\Gamma _i$ able to trace back the chemical interaction between the antagonist protein populations. In this way, the necessary feedback signals that mimic mutual recruitment, activation, and down-regulation are accounted for. In particular, the species involved here are the GPCRs and the MRPs, respectively, denoted by $\xi$ and $\zeta$. In fact, the GPCRs act as the major responsible for the activation of intracellular responses to extracellular signals (Seifert and Wenzel-Seifert 2002). In experiments, the activity of the GPCRs can be traced through the measurement of the intracellular cAMP concentrations ($cAMP_i$). For instance, in Carotenuto et al. (2020), it is extensively described the experiment on human trophoblast cells performed to detect the activity of $\beta -$adrenergic receptor. Under an incubation time of 1*h*, *cAMP* levels were measured through increasing amount of a GPCRs chemically affine ligand, namely epinephrine. Those tests showed that high level of epinephrine enhances $cAMP_i$ as $cAMP_i \approx \alpha _{\xi } \xi$, so namely there is an approximate proportionality with the GPCRs levels. On the other hand, MRPs activate after GPCRs in order to allow cytoplasmic cAMP to flow towards the extracellular environment (which will be denoted as $cAMP_e$). This control of the cell homeostasis obeys a law similar to the one above, experiments showing that $cAMP_e \approx \alpha _{\zeta } \zeta$. According to this interplay, Volterra–Lotka-like interspecific terms can be introduced and the following mass conservation equations can be written as follows:

20 $$\begin{aligned} {\left\{ \begin{array}{ll} \dot{\xi }+ \nabla \cdot \textbf{Q}_{\xi } = \xi \left( \alpha _{\xi } - \delta _{\xi } - \beta _{\xi \zeta } \zeta \right) \\ \dot{\zeta }+ \nabla \cdot \textbf{Q}_{\zeta } = \zeta \left( - \delta _{\zeta } + \beta _{\zeta \xi } \xi \right) \end{array}\right. } \end{aligned}$$

More in detail, the evolutionary dynamic of GPCRs is controlled by the activation term $\alpha _{\xi }$, which regulates the activity of the G-protein fraction in response to the ligand precipitation rate and thus strictly follows the kinetics of its space-time distribution. As in Carotenuto et al. (2020) and in accordance with experiments therein reported, this function is assumed to be temporally controlled by a generic gamma distribution with a probability density function $\gamma \left( t \right) = a t e^{-bt}$ spontaneously decaying. The ligand precipitation rate may also depend on an additional function $\eta (\textbf{x})$ describing how the ligand spatially distributes over the domain by so steering the initial arrangement of the GPCRs according to the above-mentioned proportionality. With this position, the uptake function $\alpha _{\xi }$ results to be:

21 $$\begin{aligned} \alpha _{\xi } = k_b Q^{-1} \eta (\textbf{x})\gamma \left( t \right) , \end{aligned}$$

where $k_b$ is a binding constant, while *Q* indicates the total quantity of ligand averaged over the membrane area $\Omega$:

22 $$\begin{aligned} Q=\frac{1}{\Omega }\int _\Omega \eta (\textbf{x}){\text {d}}S\int _T\gamma (t) {\text {d}}\tau = \overline{\eta }\,\int _T\gamma (t) {\text {d}}\tau . \end{aligned}$$

In the simulations below, different forms of ligand spatial fractions will be hypothesised to analyse both cases in which ligand remains compartmentalised within restricted regions of the membrane versus cases in which it randomly distributes over the membrane. For a fixed average $\overline{\eta }$, the additional parameters *a* and *b* can be calculated from experiments through the knowledge of the total concentration *Q* and experimentally observing the peak time $t_m$ at which the ligand binding is maximum, i.e. $\dot{\gamma }(t_m)=0$. The coefficients utilised here, with their units, are reported in Table 1. Moreover, in Eq. (20), the GPCRs rate is governed by the presence of the interspecific term $\beta _{\xi \zeta } \zeta$, which reproduces the down-regulating action of MRPs and by the (small) intrinsic decay rate $\delta _\xi$. With reference to the MRPs rate, activation of transporters is exclusively guided by the presence of active GPCRs through the recruitment parameter $\beta _{\zeta \xi }$, while a decay term allows for MRPs deactivation through the inhibition coefficient $\delta _\zeta$.

To gain first insights into the protein dynamics and its stability, the analysis of stationary points $n_{si}$ and related eigenvalues of the species’ Jacobian matrix $J_K^{si}$, calculated for the rates in Eq. (20), provide two possible scenarios. The first one is the extinction of the species (inactive state), i.e. $\{\xi _{s1},\zeta _{s1}\}=\{0,0\}$, with eigenvalues $\{J_{I}^{(s1)},J_{II}^{(s1)}\}=\{-\delta _\zeta ,a_\xi -\delta _\xi \}\}$. It can be readily seen that, as the ligand perturbation dies out at protracted times – i.e. $a_\xi (t\rightarrow \infty ) \rightarrow 0$ – this point becomes asymptotically stable while, as long as ligand binding is occurring and $a_\xi >\delta _\xi$, proteins deactivation is not permitted. This is consistent with the physics of the problem, since the selectivity of the membrane is strictly related to the presence of extracellular stimuli and the lipid bilayer dynamically adapt its properties during the effective signalling window. On the other hand, the second equilibrium point $\{\xi _2,\zeta _2\}=\{\delta _\zeta /\beta _{\zeta \xi }, (a_\xi -\delta _\xi )/\beta _{\xi \zeta }\}$ is related to the coexistence of active species, the associated eigenvalues in this case resulting $\{J_{I}^{(s2)},J_{II}^{(s2)}\}=\{-j\sqrt{\delta _\zeta (a_\xi -\delta _\xi )},+j\sqrt{\delta _\zeta (a_\xi -\delta _\xi )}\}$. In such a situation, when $a_\xi >\delta _\xi$ the species are in a non-equilibrium state and one would observe an oscillating interplay with (temporarily) conjugates eigenvalues, for which stability is not ensured, until signalling starts to deplete by switching to extinction. Accordingly, in this second stage, the limit condition $a_\xi (t\rightarrow \infty ) \rightarrow 0$ implies that the second critical point becomes unphysical, by returning a negative value for the MRPs fraction.

#### Stress–strain relations for a neo-Hookean membrane

Under the assumption in Sect. 2.2 of isothermal compression on the lipid system in the liquid-ordered phase, the bilayer is here supposed to behave as a hyperelastic isotropic material. Hence, a well-established hyperelastic energy helps us to mainly focus on the coupling between mechanical remodelling of the membrane and the diffusive process of the transmembrane proteins. For the sake of simplicity, the energetic tendency of lipid bilayers to organise themselves in ordered zones segregated with respect to disordered ones, even in the complete absence of proteins, is neglected (see e.g. Deseri and Zurlo (2013); Carotenuto et al. (2020) and references cited therein). To this end, a standard incompressible neo-Hookean strain energy function can be considered to model the membrane’s elastic response:

23 $$\begin{aligned} \mathcal {W}_{hyp}\left( \textbf{F}\right) =\frac{G}{2} \left( I_1-3\right) - p \left( J-1\right) , \end{aligned}$$

where $I_1=tr\left( \textbf{F}^T\textbf{F}\right)$ is the first invariant and $G=E/\left( 2\left( 1+\nu \right) \right)$ is the tangent shear modulus, while $p=p\left( x,y\right)$ is a Lagrangian pressure relaxing the isochronicity constraint. The Poisson’s ratio is $\nu \approx 0.5$ and the Young’s modulus is set to $E=10 MPa$, coherently with literature data (Janshoff and Steinem 2015). The neo-Hookean assumption is introduced in accordance with well-established evidences in the literature (Evans 1973; Evans and Hochmuth 1976; Waugh and Evans 1979) modelling the membrane as an elastic solid with this constitutive law, due to its capability to recover large deformations in response to mechanical forces. Noteworthy, imposing a finite shear modulus could appear to be in contrast with exclusively fluid behaviours (i.e. $G=0$). Notwithstanding, as well known, the low shear rigidity values of the order of $10^{-9}~{\text {N/m}}$ reported in the literature (Janshoff and Steinem 2015) can be however compatible with the possibility of exhibiting a finite shear modulus as intrinsic material constant of the bilayer, then dimensionally mediated by small geometrical quantities. Actually, given that lipid membranes show a vast variety of physical states with both liquid-like and solid-like behaviours (Espinosa et al. 2011), viscous components could be included in a straightforward manner in order to account for a liquid–solid membrane description(Evans and Hochmuth 1976). According to relations (13) and considering $J\approx 1$, the nominal Piola-Kirchhoff stress and the corresponding Cauchy stress can be calculated as follows:

24 $$\begin{aligned} \textbf{P}^*= K_r\left[ G \textbf{F} - w_i \left( n_i - n_i^0\right) \frac{\partial \phi }{\partial \textbf{F}} - p \textbf{F}^{-T}\right] , \end{aligned}$$

and

25 $$\begin{aligned} {\varvec{{\sigma }}}^*=\textbf{P}^*\textbf{F}^T = K_r\left[ G \textbf{F} \textbf{F}^T - w_i \left( n_i - n_i^0\right) (\textbf{e}_3\otimes \textbf{e}_3)\cdot \textbf{F}^T - p\textbf{I}\right] . \end{aligned}$$

As plane stress is assumed, the out-of-plane stress component $\sigma _{33}=\textbf{e}_3\cdot {\varvec{{\sigma }}}^*\cdot \textbf{e}_3$ vanishes. This returns the associated value of the Lagrangian pressure *p*:

26 $$\begin{aligned} p=G \phi ^2 - w_i \left( n_i - n_i^0\right) \phi . \end{aligned}$$

Therefore, by substituting Eq. (26) into (24), the in-plane nominal stress $P^*_0$ can be obtained:

27 $$\begin{aligned} \textbf{P}_0^* = K_r\left[ G \left( \textbf{F}_0 - \phi ^2 \textbf{F}_0^{-T}\right) + w_i \left( n_i - n_i^0\right) \phi \textbf{F}_0^{-T}\right] , \end{aligned}$$

in which one can recognise a stress of purely elastic nature and a chemical stress contribution related to the species variation (Taber 2020). Moreover, by neglecting body forces and inertia terms, the mechanical equilibrium of the membrane with respect to the active and virgin configurations reads as follows:

28 $$\begin{aligned} \nabla \cdot \textbf{P}^* = {\textbf {0}}\quad \text {or}\quad \nabla _0 \cdot \textbf{P}_0^* = {\textbf {0}}, \end{aligned}$$

with $\nabla _0$ denoting the in-plane nabla operator in the virgin configuration.

### Numerical study of the biological process

It is confirmed that GPCRs organisation on the cell membrane influences their signalling activity (Fallahi-Sichani and Linderman 2009). Such organisation has been demonstrated to be affected by different diffusion conditions present in the raft and in the non-raft domains (Woolf and Linderman 2003; Pralle et al. 2000). Those regions are known to be characterised by discrepancies in thickness membrane regulating biological functions of specific transmembrane proteins (Niemelä et al. 2007). A full description of the mechanobiological process is given through a coupling between the balance of linear momentum in (28), and the time evolution laws in (20) for the two protein fractions GPCRs and MRPs involved in the ligand binding processes, as follows:

29 $$\begin{aligned} {\left\{ \begin{array}{ll} \nabla \cdot \textbf{P}^* = {\textbf {0}} \\ \dot{\xi }+ \nabla \cdot \textbf{Q}_{\xi } - \xi \left( \alpha _{\xi } - \delta _{\xi } - \beta _{\xi \zeta } \zeta \right) = 0 \\ \dot{\zeta }+ \nabla \cdot \textbf{Q}_{\zeta } - \zeta \left( - \delta _{\zeta } + \beta _{\zeta \xi } \xi \right) = 0 \end{array}\right. } \end{aligned}$$

With the aid of the software COMSOL Multiphysics®[119], numerical solutions of Eq. (29) have been carried out by considering the model parameters reported in Table 1. In particular, a circular domain $\Omega = \{(x,y)\in R^2:x^2+y^2 \le R^2 \}$, with $R=5\mu {\text {m}}$, and a time span $t \in \left[ 0,t_{\max }\right] \}$, with $t_{\max }=1\,{\text {h}}$, have been considered in the analyses, in analogy with numerical set up referred to Carotenuto et al. (2020). The simulation, performed using the classical coefficient form PDEs, provide homogeneous initial conditions for the protein fractions $\zeta \left( x,y,0\right) = \zeta ^0$ and $\xi \left( x,y,0\right) = \xi ^0$. The in-plane displacements are both set with null initial values $\textbf{u}\left( x,y,0\right) ={\textbf {0}}$. With regard to boundary conditions, besides condition (10), null species fluxes imply the additional condition $\nabla n_i \cdot \hat{\textbf{N}} = 0$. Also, various cases have been considered in terms of mechanical boundary conditions. In particular, the results will refer to: (i) a fully constrained case, in which $\textbf{u}\cdot \hat{\textbf{N}}={\textbf {0}}$ at the external radius; (ii) a dual traction-free case, where vanishing stresses at the boundary have been accounted for $\textbf{P}^* \cdot \hat{\textbf{N}}= {\textbf {0}}$; (iii) for some other cases, stress-prescribed situations have been considered, where a nonzero radial stress at the boundary is imposed to simulate the membrane Laplace tension due to intracellular pressure. In particular, the nominal traction in the radial direction at the outer radius, i.e. $\textbf{P}^* \cdot \hat{\textbf{N}}= T_R\hat{\textbf{N}}$, can be evaluated by starting from a prescribed outer (actual) pressure $p_o$, so that the equivalence $p_o\,h\,ds=T_R\,h_0\,{\text {d}}S^0$ leads to $T_R=p_o(1+u_R/R)/J_0$, $u_R$ denoting the modulus of the in-plane displacement at the boundary.

**Table 1:** Summary of the numerical values for the coefficients entering in the model. Some of them are well known in the literature, while others have been assumed in certain ranges after thousands of numerical simulations

Coefficient	Value [Unit]	Range [Unit]	Reference
$L_i$	$7\times 10^{-17}[{\text {m}}^{2}{\text {Pa}}^{-1}{\text {s}}^{-1}]$	$\left( 10^{-20}-10^{-15}\right) [{\text {m}}^{2}{\text {Pa}}^{-1}{\text {s}}^{-1}]$	Carotenuto et al. (2020)
$k_b$	5.18	$3.89-5.7$	Bridge et al. (2018); Li et al. (2017)
*Q*	$2000 [{\text {pMol}}]$		Carotenuto et al. (2020)
$\delta _{\xi }$	$1.1 \times 10^{-3} [{\text {s}}^{-1}]$	$\left( 0.9-1.65\right) \times 10^{-3} [{\text {s}}^{-1}]$	Bridge et al. (2018)
$\delta _{\zeta }$	$10^{-7} [{\text {s}}^{-1}]$	$\left( 10^{-8}-10^{-6}\right) [{\text {s}}^{-1}]$	Carotenuto et al. (2020)
$w_{\xi }$	$5.25[{\text {MPa}}]$	$\left( 5-8\right) [{\text {MPa}}]$	Carotenuto et al. (2020)
$w_{\zeta }$	$2.25[{\text {MPa}}]$	$\left( 2.17-3.5\right) [{\text {MPa}}]$	Carotenuto et al. (2020)
$\beta _{\xi \zeta }$	$1.25\times 10^{-2}[{\text {s}}^{-1}]$		–
$\beta _{\zeta \xi }$	$1.28\times 10^{-2}[{\text {s}}^{-1}]$		–
$\xi ^0$	$10^{-1}$		–
$\zeta ^0$	$10^{-2}$		–

## Results and discussion

Within the presented framework, numerical solutions allow us to investigate the role that mechanics plays in the spatio-temporal dynamics of the raft-associated proteins and the adaptive remodelling of the bilayer under the activation of the GPCRs in the various configurations considered in this formulation. This is in order to theoretically elucidate some coupling mechanisms behind lipid membrane organisation and functionality. Through simulations and ad hoc sensitivity analyses, the focus here is on studying how the interplay of mechanical and biological factors can explain the experimentally observed preference of active GPCRs in co-localising and clustering on lipid islands. This can be done by examining the mechanical influence in the coalescence of protein micro-domains in terms of conformational changes and mobility. In turn, this has a direct effect on membrane thickness re-arrangement in various scenarios and under different mechanical boundary conditions.

**Fig. 3:**
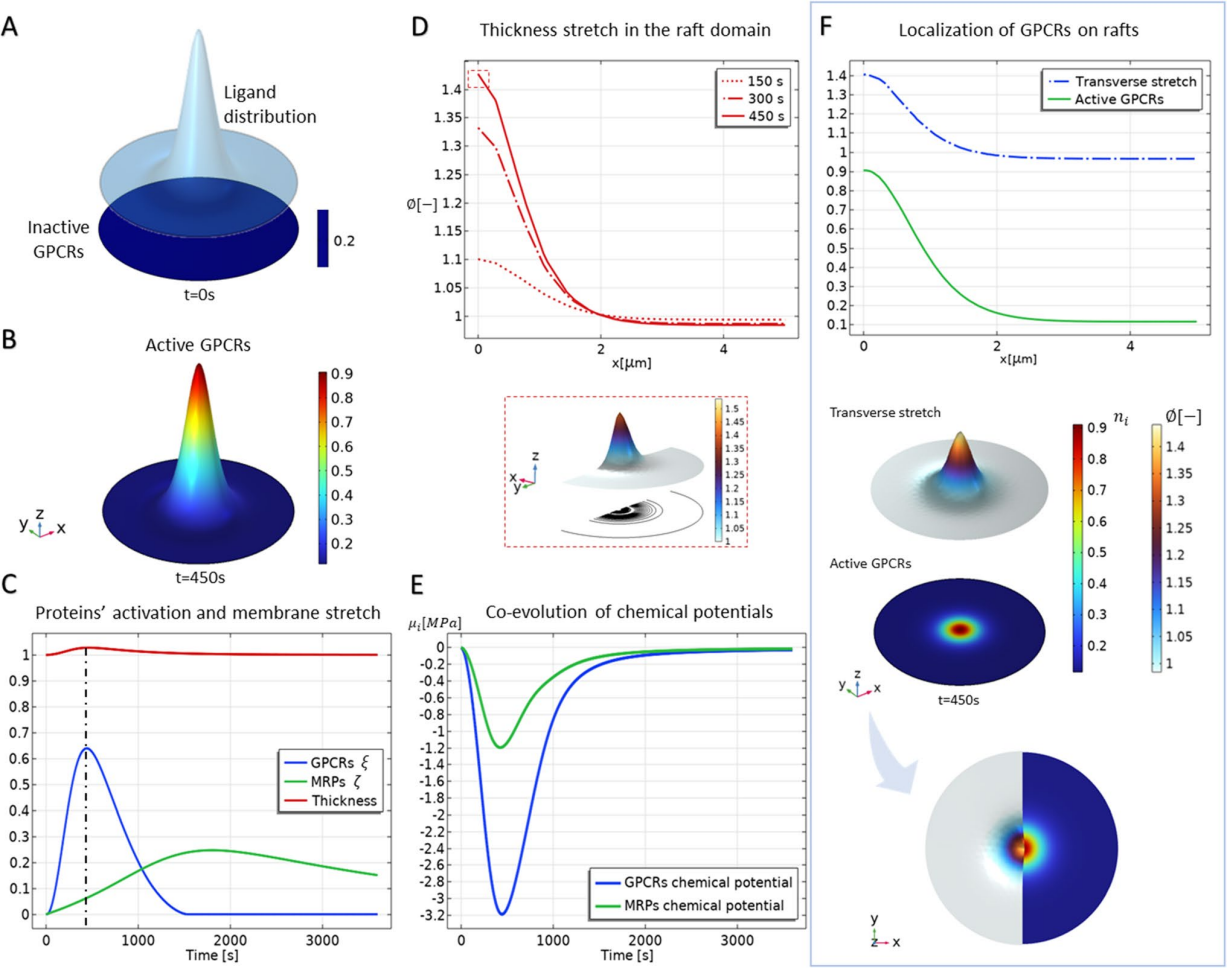
Co-localisation of active protein domains on single raft formation in a confined membrane. **A**: Initial configuration of inactive GPCRs (blue) and ligand distribution (light blue) at time $t=0~{\text {s}}$. **B**: Active protein species domain at time $t=450~{\text {s}}$ where it is maximum. **C**: Evolution over time of the interspecific dynamics, displayed in terms of area-averaged quantities. The synergistic activation of GPCRs with the formation of raft domains is here observed passing from protein inactive state at $t=0~{\text {s}}$ to protein activation at $t=450~{\text {s}}$. The activation of MRPs later occurs. **D**: Progressive rafts formation in the region where active GPCRs tend to cluster. **E**: Time evolution of the area-averaged chemical potentials. **F**: Correlation among the active protein fraction and the membrane deformation at time $t=450~{\text {s}}$

***Influence of chemo-mechanical interplay*** In the proposed approach, the spatio-temporal variation of transmembrane protein densities can actually be translated into an effective remodelling of the lipid bilayer in terms of lipid order and accumulation of in-plane pressure induced by the conformational changes of the protein structures. In this way, we aim at highlighting the primary role of chemo-mechanical coupling in the formation of raft domains. On the other hand, re-configuration of membrane thickness constitutes a mechanotaxis stimulus for active species mobility and recruitment. In the model, this feedback mechanism is fulfilled by the coupling terms mediated by the chemo-mechanical terms $w_i$ (see Table 1), standing for the work done by the lateral pressure to confine the transmembrane proteins of the *i*${\text {th}}$ species while allowing their conformational changes across the thickness of the bilayer. This permits us to consider both density-induced stresses and raft-induced attraction (see Eqs. (27) and (19), respectively). With this in mind, a single-raft pilot study is carried out by assigning a normal distribution to the spatial probability density $\eta (\textbf{x})$ of precipitating ligands, centred in the middle of the domain $\Omega$ (see Fig. 3A). Such a position affects the uptake function $\alpha _{\xi }$, activating the GPCRs response to ligands by thus triggering the protein dynamics. The results in Fig. 3B show that the activation of the ligand–receptor complexes reaches its maximum value at time $t=450~{\text {s}}$, as also highlighted in Fig. 3C, where the temporal profiles of G-proteins and receptors are displayed to emphasise their interspecific correlation. By observing the obtained dynamics, it can be seen how GPCRs initially grow while binding and starting the signalling. The dynamical activation of GPCRs induces a subsequent triggering of cellular mechanisms that leads to the gradual involvement of the MRPs, which desensitise receptors before decaying. The obtained receptor density profiles are also compatible with the persistence times of intracellular and extracellular cAMP concentrations used in experiments to trace and detect the activity of $\beta$-adrenergic receptors and transporters (Carotenuto et al. 2020). Figure 3C displays the simultaneous evolution of the ordered lipid phase, for which the membrane thickness achieves its maximum at $t\approx 450~{\text {s}}$, precisely at the highest expression of active proteins’ overall population. Such dynamics is consistent with the short lifetime of lipid rafts observed in the literature (Sezgin et al. 2017; Kusumi et al. 2011), experiments showing how ordered islands disappear after the ligand binding annihilates due to depletion of the binding molecules by then relaxing in thickness and recovering the physiological condition. It is worth noticing that the obtained kinetics of Fig. 3C results slower than the characteristic times reported for the activation mechanisms of single GPCR molecules (Grushevskyi et al. 2019), the simulations here referring to the areal-averaged response of the entire GPCRs’ population the prescribed ligand precipitation. Furthermore, as shown in Fig. 3D, the evolution of the lipid raft is backed by the progressive increase of the transverse stretch along the radius, the deformation and the species evolution concurring in the mechanical stress and chemical potential within the raft domain (see Fig. 3E). In agreement with thermodynamical compatibility, the chemical potentials turn out to achieve negative values in the presence of the binding kinetics. To highlight the effect of co-localisation, membrane thickening, and active GPCRs are directly compared in Fig. 3F, which displays how the single raft actually co-occurs with the binding/unbinding kinetics of the active transmembrane proteins.

**Fig. 4:**
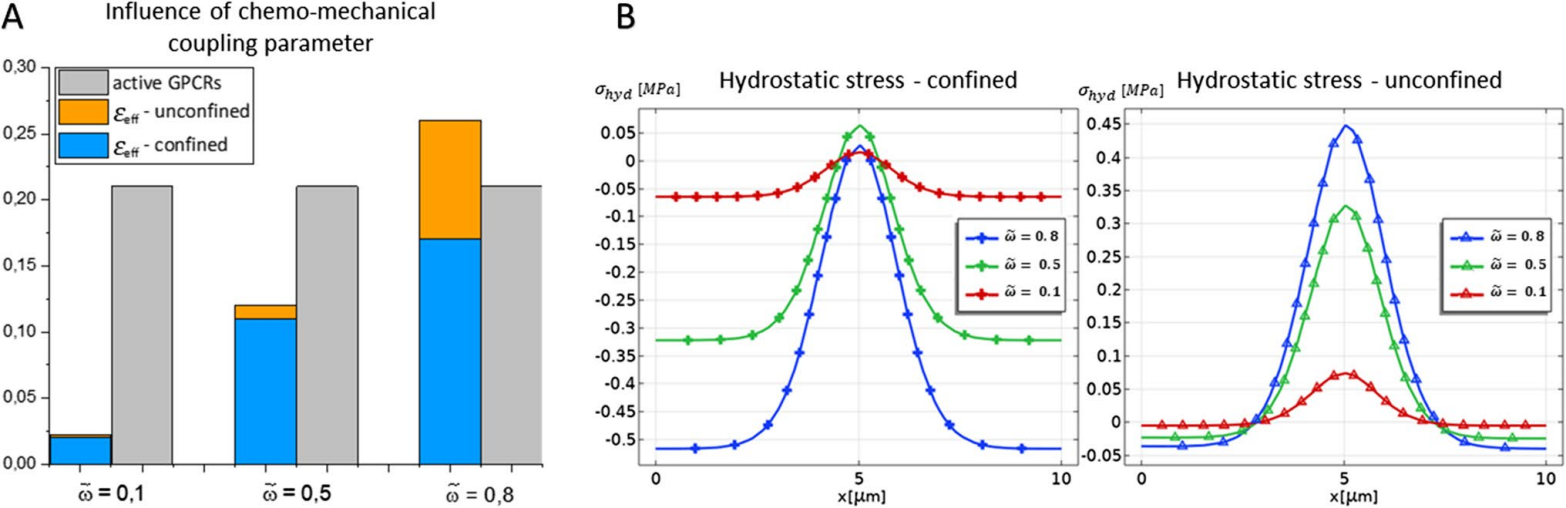
**A**: Influence of the chemo-mechanical interaction parameter $\tilde{w}$ on ordered lipid phase, measured in terms of effective strain as the relative height variation between ordered and disordered phases, i.e. $\varepsilon _{\text {eff}}=h_{{\text {ord}}}/h_{{\text {dis}}}-1$. **B**: Influence of the chemo-mechanical interaction parameter $\tilde{w}$ on hydrostatic stress profiles in confined and traction-free cases. Three curves are obtained by varying the chemo-mechanical parameter $\tilde{w}$

Noteworthy, the process of rafts formation seems to be dominated by the ligand binding trigger, the deformability of the membrane resulting to not significantly affect the membrane thickening. The influence of chemo-mechanical coupling on the dynamics described above is summarised in Fig. 4. Herein, a sensitivity analysis has been performed by varying the weight of the coupling parameters $w_\xi$ and $w_\zeta$ with respect to the elastic modulus of the membrane (specifically, we introduce the parameter $\tilde{w}=w_\xi /E$, while the ratio $w_\xi /w_\zeta$ is kept fixed). This variation corresponds to considering the possible different capabilities of activating receptors to exchange lateral pressure with the membrane while re-configuring their structures. This means that the micro-mechanical work per unit area and per unit receptor can vary according to microscopic interactions between the protein and surrounding lipids. Such work essentially conveys the idea of how much effort is made by protein structures to rearrange themselves within the lipid bilayer (a micro-mechanical interpretation of these parameters is detailed in Carotenuto et al. (2020)). In the macroscale study at hand, it can be appreciated how coupling highly affects the mechanical micro-environment. As reported in Fig. 4A, these specific coupling coefficients indeed result not to affect the amount of GPCRs, whose activation is mostly driven by the availability of chemically affine binding molecules. However, their feedback on the surrounding membrane highly differs from case to case when $w_i$ changes. In particular, Fig. 4A suggests that raft formation is enhanced as the interaction coefficient $\tilde{w}$ increases. As highlighted, this effect intensifies in the unconfined situation, where the membrane deforms more freely while exhibiting a higher effective thickness. Correspondingly, in Fig. 4B, chemo-mechanical coupling and boundary conditions affect the hydrostatic stress distribution, i.e. $\sigma ^*_{\text {hyd}}=\left( \sigma ^*_{11}+\sigma ^*_{22}\right) /2$, in the membrane. Indeed, in both cases, a weak coupling does not produce significant stresses within the raft domain, while the exerted pressure grows when $\tilde{w}$ approaches 0.8. Furthermore, in the confined membrane, raft formation induces an approximately homogeneous compression in the surrounding disordered phase that deepens as the level of the interaction increases.

**Fig. 5:**
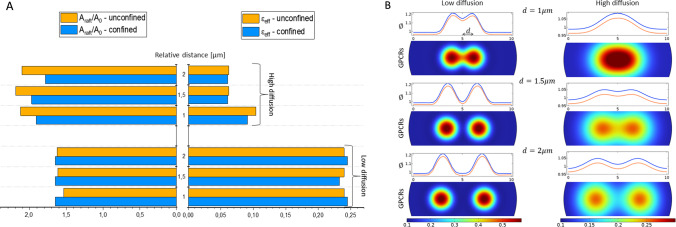
Effect of species diffusivity on raft morphology. **A**: Comparison of effective areal extension $A_{\text {raft}}/A_0$ and effective height strain $\varepsilon _{\text {eff}}$ in low diffusion $\left( \epsilon , \gamma \right) =\left( 0.05~{\text {Pa}}^{-1},0.1~{\text {Pa}} \,\upmu {\text {m}}^2\right)$ and high diffusion $\left( \epsilon , \gamma \right) =\left( 20~{\text {Pa}}^{-1},10~{\text {Pa}} \,\upmu {\text {m}}^2\right)$ cases. **B**: Comparison, at the maximum activity time $t=450~{\text {s}}$, of raft morphology in terms of active protein concentrations and corresponding thickness variation

***Role of diffusion in raft formation*** Species mobility and sensitivity to relative position of protein clusters is a further aspect investigated to gain some insights into the mechanism by which active receptors exchange chemo-mechanical signals. This is done by determining mutual attraction and so favouring nucleation of macro-islands. In this sense, chemical diffusion has a direct role in steering the spatial organisation of active proteins. To explore this aspect, we considered a second paradigm in which ligand precipitation occurs in two different spots at a varying distance between each other and by further reproducing two different scenarios: a case of low diffusion and a case of high species diffusion, carried out by calibrating the parameters $\epsilon$ and $\gamma$ in Eq. (19). In Fig. 5A, we examine the effect of diffusion on the morphological features of the raft, namely the total relative area occupied by the raft (at the maximum time $t\simeq 450~{\text {s}}$) and the relative height variation. Low diffusion potentials produce higher concentrated active species in a smaller area $A_{{\text {raft}}}$, although the latter enlarges with respect to the initial spot areas $A_0$ by about the $50\%$. If the motility of the species is enhanced, the produced raft islands appear more extended in the membrane plane and thinner, due to the fact that the same amount of proteins interacts with the surrounding lipids by distributing itself over a larger region. From a spatial viewpoint, as reported in Fig. 5B, in low diffusive simulations the emerging rafts do not communicate and evolve almost independently, except for the case in which the spots are at a total distance of $2~~\upmu {\text {m}}$ one from the other. On the other hand, in the highly diffusive scenario, the coalescence is always appreciated although its effect decreases as the relative distance increases, as the last inter-distance among the spots of $4~\upmu {\text {m}}$ that produces a highly feeble interaction. However, in the dynamics at hand, pure coalescence of the active domains cannot be followed completely since GPCRs are gradually de-activated by the competing action of transporters.

**Fig. 6:**
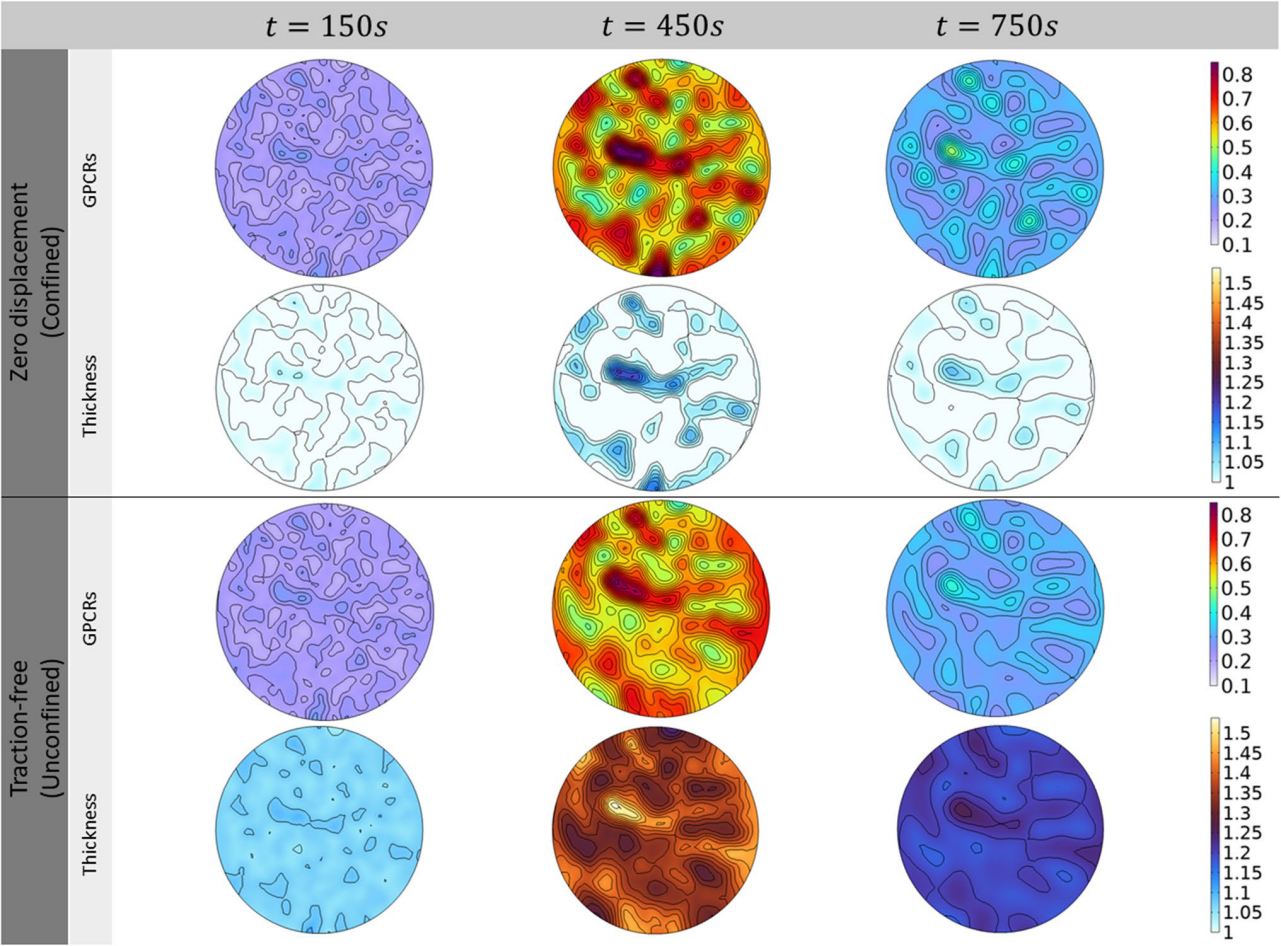
Surface plots of the active GPCRs domains localised on lipid rafts. Membrane thickening is inspected through strain measures associated with the activation of the protein fractions in a confined configuration above and in a traction-free configuration below. At time $t=450~{\text {s}}$ the maximum concentration of active GPCRs is reached and also the maximum thickening is observed. Then, the membrane relaxation occurs at time $t=750~{\text {s}}$

**Fig. 7:**
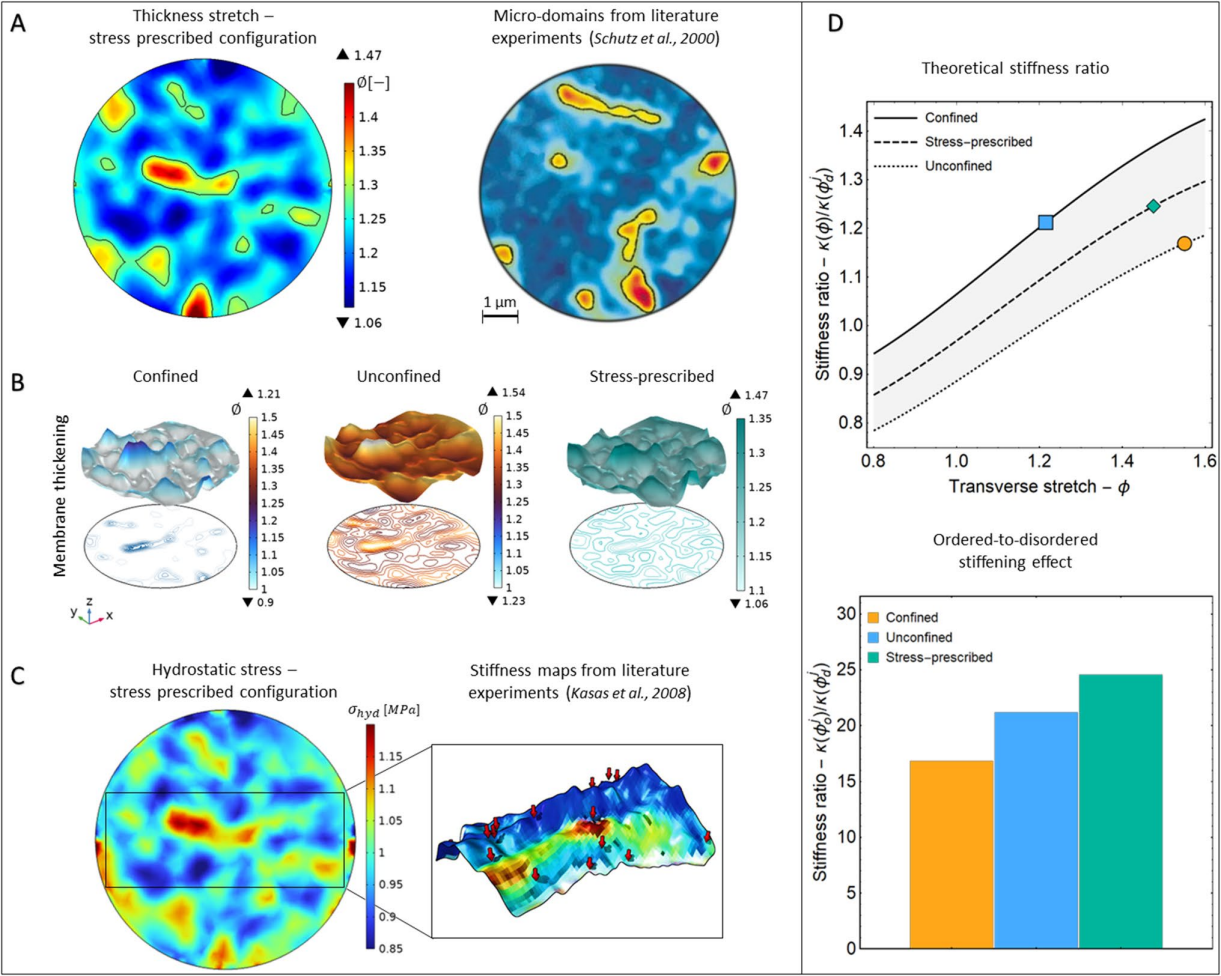
**A**: (*Left*) Thickness variation in stress-prescribed conditions and random precipitation. (*Right*) Patterning of micro-domains obtained from literature experiments (Schütz et al. 2000). **B**: Comparison of thickness variation in confined, unconfined and stress-prescribed conditions with random ligand precipitation. **C**: Theoretical prediction of hydrostatic stress in qualitative comparison with the stiffness maps obtained in Kasas et al. (2008). **D**: (*Top*) Theoretical stiffening ratio as a function of the transverse stretch and (*Bottom*) comparison of ordered-to-disordered stiffening values in comparison with the experimentally observed stiffening range

***Morphological and material remodelling in actual membranes*** In order to have a deeper insight into the co-localisation of active domains on lipid rafts, we consider a more realistic situation in which extracellular molecules randomly precipitate on the membrane area. This is done by assigning a random distribution to the ligand rate function in Eq. (21). For these simulations, the formation of lipid rafts can be depicted by following the co-evolution of active receptors and membrane thickness in Fig. 6. Therein, at different times, the initially inactive proteins start to uptake ligands by segregating to form active macro-complexes that, by means of coupling mechanisms, give rise to membrane re-arrangement and raft emergence. In particular, starting from the same initial conditions, both the cases of confined and unconfined domains are analysed. At time $t=150~{\text {s}}$, the activation and the subsequent densification of the GPCRs are accompanied by progressive coalescence. Those are detected by the strain localisation on thicker zones witnessing liquid-ordered phase transition and forming lipid islands. Again, their maximum value is found at $t=450~{\text {s}}$. After that, the unbinding kinetics lead to a lower concentration of GPCRs and membrane relaxation at $t=750~{\text {s}}$. As shown in Fig. 6, in confined coalescence lipid membranes exhibit a maximum transverse stretch of 1.22, while the raft islands appear more prominent in stress-free conditions, where the observed rafts have an increase of more than the $50\%$ in height. In order to mimic more realistic cell situations, the membrane can experience intermediate mechanical conditions by perceiving a nonzero membrane tension. This is due to the force exchange with the intracellular environment during various cell processes such as spreading, adhesion, or proliferation. In such a more realistic case, a stress-prescribed case has been considered as an additional boundary condition, as already discussed in Sect. 2.3. The results are synoptically represented in the panel of Fig. 7. More in detail, Fig. 7A displays the predicted ordered domains obtained by starting from initial conditions analogous to the previous case. Noteworthy, these numerical outcomes well reproduce some experimental patterns observed in the literature (see e.g. Schütz et al. 2000; Lu et al. 2008), in which different techniques were used to detect the formation of micro-domains of ordered lipids in cell plasma membranes. In Fig. 7B, a direct comparison between the three boundary conditions is carried out, the stress-prescribed case inducing a thickness change slightly lower than the unconfined case. Importantly, in the realistic stress-prescribed case and in the unconfined one, the out-of-plane membrane thickening is up to about the $40\%$, while does not go below 1.06. Volumetric incompressibility suggests that membrane re-arranges by avoiding in-plane expansion, the reciprocal areal change experiencing contraction levels ranging from the $6\%$ to about the $30\%$. This is consistent with literature evidences showing that plasma membranes are poorly extensible systems, showing a rupture (expansion) strain varying from about $3\%$ to $6\%$ (Le Roux et al. 2019). On the other hand, the fully confined case shows zones with areal expansions approaching approximately the $10\%$, suggesting the occurrence of possible critical conditions induced by the excessive external constraint. Also, in all the cases, the obtained height turns out to be compatible with experimental observations reporting that lipid rafts are 1–$2~{\text {nm}}$ higher than the surrounding membrane (the reference thickness of a bilayer being about $5~{\text {nm}}$) (Yuan et al. 2002; Zaborowska et al. 2021),130. The direct coupling with the mechanical problem allows us to estimate the in-plane membrane stresses. The hydrostatic pressure, reported in Fig. 7C, shows how active domains are accompanied by higher lipid stresses. This is expected to have a direct impact on the membrane remodelling in terms of stress-induced heterogenisation of material properties with possible localised stiffening (Carotenuto et al. 2019). Importantly, literature findings actually indicate that lipid rafts result on average $30\%$ stiffer than non-raft regions, this datum deriving from the direct investigation of cell membrane elastic properties by means of atomic force microscopy in several studies (Roduit et al. 2008; Kasas and Dietler 2008; Kasas et al. 2008; Et-Thakafy et al. 2019). In the present model, the evaluation of stresses occurring in correspondence of raft domains qualitatively suggests a strong correlation with the relative stiffening measured during indentation experiments (see Fig. 7C). In order to provide a straightforward validation of the numerical outcomes, the relative stiffening between ordered and disordered phases, dependent on the theoretically predicted stresses, has been calculated thanks to analytical estimations deriving from well-established literature results, as explicitly reported in Appendix. In Fig. 7D, this stiffness ratio $\kappa (\phi _o)/\kappa (\phi _d^{j})$ has been plotted as a function of the transverse stretch: the corresponding curves obtained in the different cases show a monotonic growth with respect to the raft thickening. Furthermore, for the predicted thickenings of raft domains compared to those ones of the surrounding membrane (see Fig. 7D), the ordered-to-disordered stiffening ratios turn out to be also quantitatively compatible with the experimental range determined by mechanical tests. There, the stress-prescribed case actually shows the highest stiffening ratio approaching the $\approx 25\%$, in good agreement with literature findings.

## Conclusions

The presented numerical study allows to confirm that active GPCRs transmembrane proteins localise on lipid rafts. Thanks to the described full-coupling, the in-silico analyses helped to highlight the tendency of GPCRs regions to coalesce and cluster on such islands, a fact that has been experimentally demonstrated in the literature. By enriching the well-established multiphysics approach in Carotenuto et al. (2020) to model the kinetics of phase transitions and raft emergence, combined with the mechanical work that the activated proteins exert on the surrounding lipid membrane and their tendency to coalesce, a quantitative predicting model is here obtained. The latter is capable of following the highly dynamic context of the heterogeneous membrane activity by reproducing both morphological re-organisation of lipid phases and the associated remodelling of material properties due to internal stress re-distribution.

Various scenarios have been shown for characterising the space-time variations of rafts domains within the cell membrane. Outcomes successfully aim at observing the co-localisation and synergy between the activation of these proteins and raft formation. Moreover, the results from these complex interspecific dynamics exhibited different morphological arrangements due to alterations in diffusive walkways and coalescence phenomena, that enhance the growth of macro-domains.

In particular, sensitivity analyses revealed how the combination of enhanced diffusive dynamics and finite elasticity—although in its simplest form—can be utilised to predict membrane functionality in the presence of active species and adaptation in homeostasis as well as in states away from that. To obtain a more faithful membrane characterisation, a visco-hyperelastic model could be considered by adding viscosity to capture the influence of fluidity of the lipid phase, although the estimated characteristic relaxation times of the order of units of milliseconds (Espinosa et al. 2011) would, at least in theory, induce creep phenomena at time scales lower than the characteristic rates governing the activation of protein species in the presented dynamics ($\propto$10–100 s). Thanks to inter-specificity, it is possible to include various agents determining the membrane dynamics. This could let to include several receptor species not only operating via cAMP pathway and the interaction with different binding molecules. Various chemicals interacting within a multi-species environment could produce enriched scenarios, where the role and the effect of each molecular species can be taken into explicit account. This could have a potential impact on the possibility of directly influencing some key mechanisms at the basis of communication between the cell and its surrounding extracellular environment. Furthermore, the present approach may be beneficial for studying alterations of normal cell pathways and gene expression, enabling the comprehension of the mechanisms behind cellular membrane alteration and re-configuration. To this aim, such complex multi-species environment could be enriched by including in the membrane dynamics other important components that participate in the formation and stabilisation of lipid rafts. This will be object of future investigations.

## Data Availability

Not applicable.

## Appendix

### Expression of the theoretical indentation stiffness

The expression of the ordered-to-disordered stiffening ratio has been obtained by referring to well-established literature results (Zisis et al. 2015). In particular, in order to reproduce the effect of AFM probing, the focus is on considering the indentation of a nonlinearly elastic substrate, which in this case obeys a volume-preserving neo-Hookean law. Under these assumptions, by adopting a small-on-large approach, Green et al. (1970) analytically derived a modified indentation relation that reads as follows:

A1 $$\begin{aligned} F_i= \frac{16}{9}E\,\chi (\lambda _p)\,d_i^{3/2}R_i^{1/2} \end{aligned}$$

where $F_i$ is the indentation force, $d_i$ is the indentation depth, $R_i$ is the indenter tip radius, while the tangent Young modulus *E* is weighted by a stretch-dependent factor $\chi (\lambda _p)$. For incompressible neo-Hookean substrates with an initially equi-biaxial in-plane stretch state, such factor has the following expression:

A2 $$\begin{aligned} \chi (\lambda _p)=\frac{\lambda _p^9+\lambda _p^6+3\lambda _p^2-1}{2\lambda _p^4(1+\lambda _p^3)}. \end{aligned}$$

In our model, as a first approximation, numerical solutions show that the emergence of raft domains acts in-plane as *local centre of contractions*, in which the principal stretches are quantitatively similar to each other. Thus, a simplifying hypothesis on the deformation state leads to consider $\lambda _{01}\simeq \lambda _{02} \simeq \lambda _p$ and $\lambda _p=1/\sqrt{\phi }$ locally.

Furthermore, the above analytical solution can be adapted to the case of indentation of a thin layer of hyperelastic material that rests on a rigid ground. To account for this and assuming indentation as a small perturbation, a further approximation of the Hertz solution is used by replacing the indentation depth with an equivalent indentation depth $d_i^{eq}$ respecting the equation (Dimitriadis et al. 2002):

A3 $$\begin{aligned} \frac{d_i}{d_i^{eq}}={} & {} 1 + 0.884 \left( \frac{a_h}{h}\right) + 0.781 \left( \frac{a_h}{h}\right) ^2 + 0.386 \left( \frac{a_h}{h}\right) ^3 \nonumber \\{} & {} + 0.0048 \left( \frac{a_h}{h}\right) ^4; \end{aligned}$$

where $a_h=\sqrt{R_i\,d_i^{\text {eq}}}$ and, in our case, the thickness *h* locally varies at each indentation point, i.e. $h=\phi \,h_0$. By considering that $d_i\approxeq \epsilon R_i$ and by substituting values compatible with those used in literature experiments ($R_i=20~{\text {nm}}$, $d_i\le 50~{\text {nm}}$ and $h_0=4-5~{\text {nm}}$ (Roduit et al. 2008)), the stiffness estimations as a function of the transverse stretch have been obtained by deriving of Eq. (A1) and by accounting for relation (A3).

## Code availability

All code for data analysis associated with the current submission is available under reasonable requests.
